# Supplementary material for: Hypothalamic CRH neurons represent physiological memory of positive and negative experience
Source: Nat Commun. 2023 Dec 21;14:8522. doi: 10.1038/s41467-023-44163-5 (PMC10739955; doi:10.1038/s41467-023-44163-5)
Supplement: Supplementary file 1 — Supplementary Information [file 41467_2023_44163_MOESM1_ESM.pdf]

Hypothalamic CRH neurons represent physiological memory of positive and negative experience

Supplementary Figures

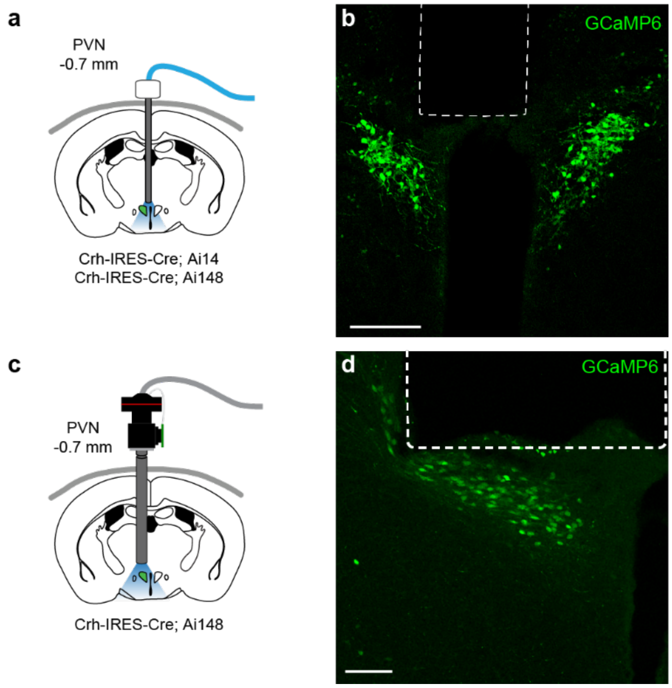

Supplementary Fig. 1. Implantations for fiber photometry and miniature microscopy.
a, Schematic illustration of ferrule placement. **b**, Low magnification confocal image shows the fiber photometry ferrule implantation site (dashed line) dorsal of the paraventricular nucleus of the hypothalamus (PVN). **c**, Schematic illustration of GRIN lens placement. **d**, Low magnification confocal image shows the lens implantation site (dashed line) dorsal of the PVN. Scale bar, **b**, 200 µm, **d**, 100 µm.

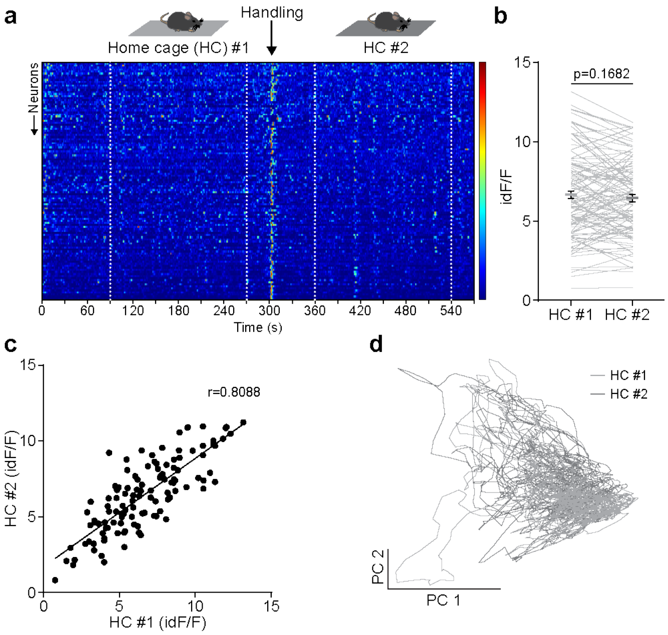

Supplementary Fig. 2. Impact of handling on CRH^{PVN} activity.

a, Heat maps show the activity of identified neurons before (#1) and after (#2) handling in the home cage (HC). **b**, Activity of identified units during HC#1 and HC#2 (p=0.1682, n=116 cells, N=4 animals, t=0.1387, paired two-tailed t-test). **c**, Correlation of neuronal activity between HC#1 and HC#2 ($p<10^{-15}$, r=0.8088, linear regression). **d**, Principal component analysis result shows the relationship of neuronal activities between HC#1 and HC#2. Data are mean ± s.e.m. Scale bar, **a**, 0-100 idF/F.

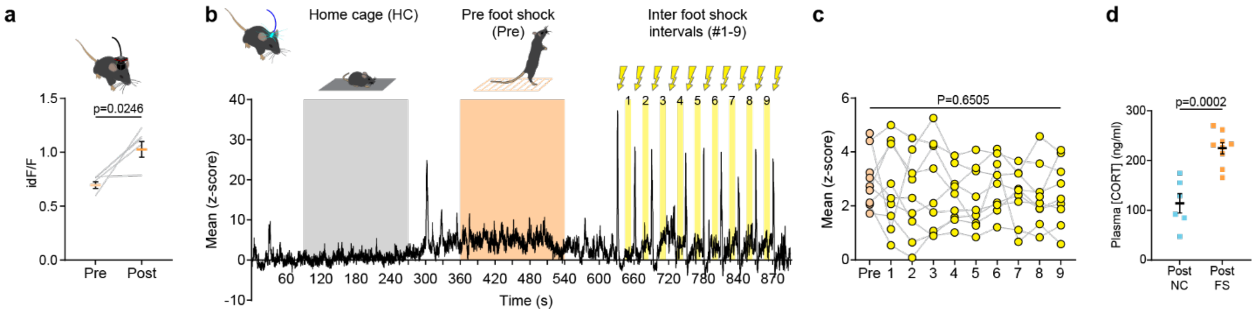

Supplementary Fig. 3. Population activity of CRH^{PVN} neurons upon exposures to foot shocks and foot shock context.

a, Activity of identified units pooled by animals neurons during Pre and Post (p=0.0246, n=5 animals, t=3.515, paired two-tailed t-test). **b**, Illustrative fiber photometry trace showing the 3 min-long time segments of home cage (HC) and context (Pre) exposures before foot shock, as well as the 10 sec-long segments between foot shocks used for further analysis. **c**, The mean of CRH^{PVN} neurons population activity before and between repeated foot shocks (P=0.6505, n=9 mice, F(9,72)=0.7630, repeated measures 1-way ANOVA). **d**, Corticosterone response to re-exposure to neutral (NC) and foot shock (FS) context (p=0.0002, t=5.276, two-tailed t-test). Data are mean ± s.e.m.

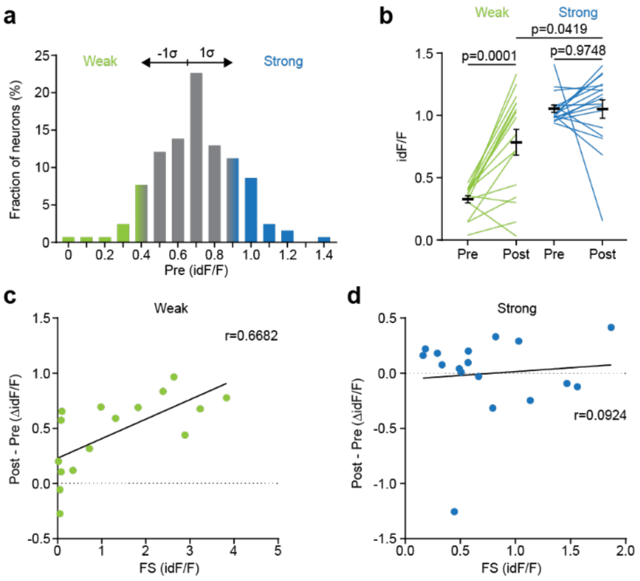

Supplementary Fig. 4. Clustering of CRH^{PVN} neurons based standard deviation.

a, Distribution of CRH^{PVN} activity. Neurons with an anticipatory activity more than 1σ away from the mean were grouped as Weak and Strong anticipator neurons. **b**, Neuronal activity in Pre and Post of Weak and Strong anticipatory neurons (Weak, $p=1.076x10^{-4}$, n=16 cells, t=5.201, paired two-tailed t-test); (Strong, p=0.9748, n=17 cells, t=0.0320, paired two-tailed t-test). Comparison whether the subpopulations are still distinguishable on Post (p=0.0419, t=2.122, two-tailed t-test). **c**, Correlation between foot shock (FS) and the subsequent activity increase observed on Post of Weak neurons (p=0.0047, r=0.6682, linear regression). **d**, Correlation between FS and the subsequent activity increase observed on Post of *Strong* neurons (p=0.7244, r=0.0924, linear regression). Data are mean ± s.e.m.

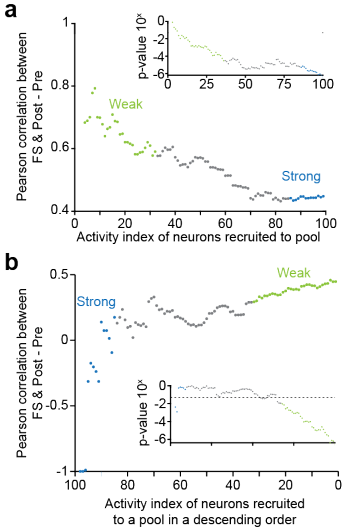

Supplementary Fig. 5. The level of anticipatory activity defines the correlation between the foot shock response and the activity update.

a, Plot of Pearson correlation results between foot shock (FS) and subsequent CRH^{PVN} activity is estimated between the population of neurons that have the *k*th percentile of anticipatory activities for increasing *k*. Inset shows the calculated p values of the correlations. Colors indicate the inclusion of neurons formerly clustered as Weak and Strong anticipatory neurons. **b**, Same correlation plot as neurons are included in the analysis in a descending order based on anticipatory activity. Inset shows the calculated p values of the correlations.

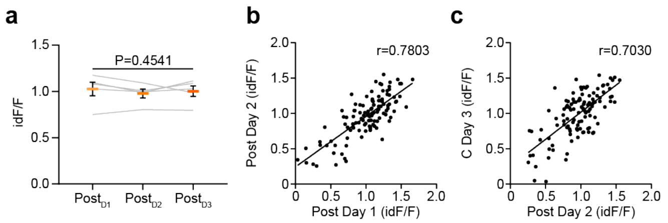

Supplementary Fig. 6. Day-to-day neuronal activity correlations upon repeated exposures to foot shock. **a**, Activity of identified units pooled by animals is similar during all repeated exposures to the foot shock context (P=0.4541, n=5 mice, F(2,8)=0.8726, repeated measures 1-way ANOVA). **b**, Correlation of neuronal activity between Post$_{Day\ 1}$ and Post$_{Day\ 2}$ ($p<10^{-15}$, r=0.7803, linear regression). **c**, Correlation of neuronal activity between Post$_{Day\ 2}$ and Post$_{Day\ 3}$ ($p<10^{-15}$, r=0.7030, linear regression). Data are mean ± s.e.m.

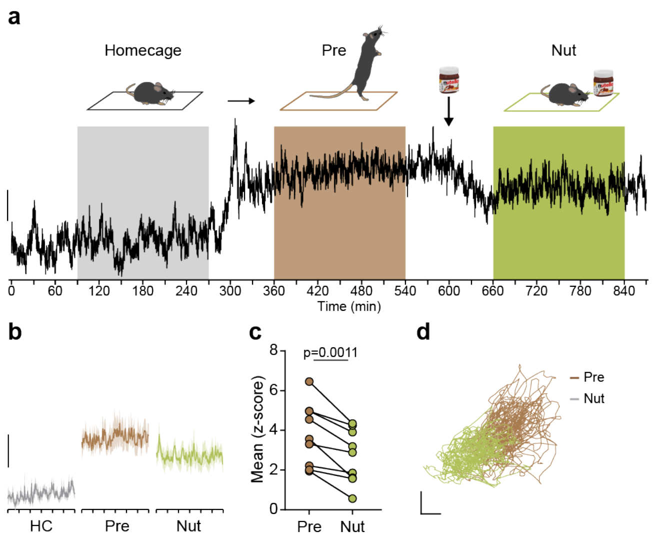

Supplementary Fig. 7. Immediate and contextual effects of appetitive stimulus on the CRH^{PVN} population activity.

a, Illustrative fiber photometry trace showing the 3 min-long time segments of home cage (HC), the context prior the presentation of the hazelnut spread (Pre) and the presentation of the spread in the context (Nut). **b**, Mean traces during HC, Pre and Nut. **c**, Quantification of the mean amplitudes during the different states (p=0.0011, n=9 mice, t=4.952, paired two-tailed t-test). **d**, Principal component analysis of CRH^{PVN} activity before and during hazelnut exposure in the neutral context. Scale bars, **a**, **b**, 2 z-score. Data are mean ± s.e.m.

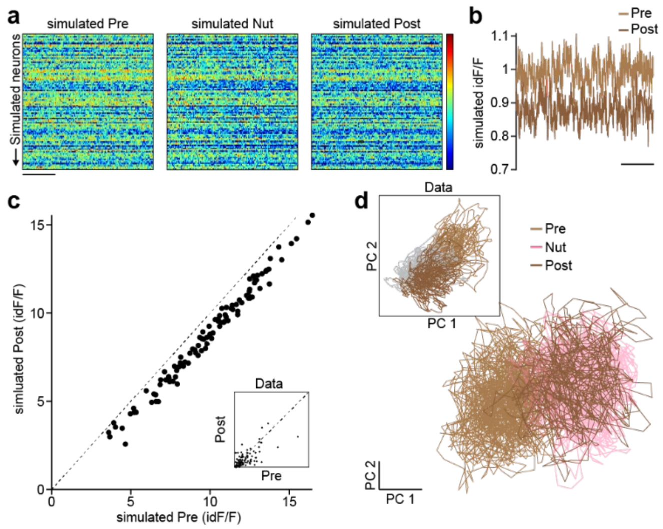

Supplementary Fig. 8. Updating rule for endocrine appetitive memory.
a, Simulated calcium indicator in the Pre, Nut, and Post. **b**, The simulated calcium indicator decreases on Post. **c**, Change in simulated calcium indicator variable (with data inset). **d**, Principal component analysis plots of the simulated network (with data inset). Scale bar, **a**, **b**, 30 s.

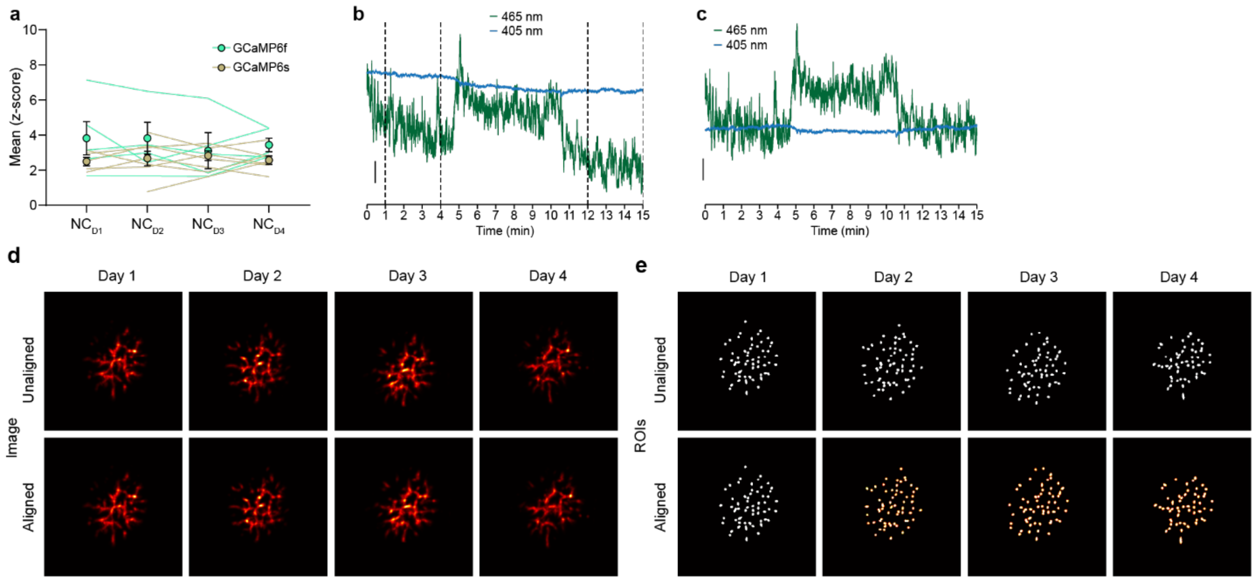

Supplementary Fig. 9. Fiber photometry data analysis and longitudinal alignment of neurons recorded by miniature microscopy.

a, Mean amplitude recorded during repeated exposure to a neutral environment using the expression of GCaMP6s or GCaMP6f in CRH^{PVN} neurons (P=0.2015; F(1,10)=1.870; mixed effects two-way ANOVA). **b**,**c**, Fiber photometry traces before (**b**) and after (**c**) bleaching correction show the raw recording of the GCaMP signal (465 nm, red) and the control channel (405 nm, blue) used for motion correction. The section between the dashed lines shows the time period used for bleaching correction. **d**,**e**, Neuronal alignment is based on the summarized images and the regions of interest (ROIs) provided by Min1pipe. **d**, Summary images before (top) and after (bottom) alignment. **e**, Selected ROIs before (top) and after (bottom) alignment. Scale bars: **b**, **c**, 20 mV. Data are mean ± s.e.m.

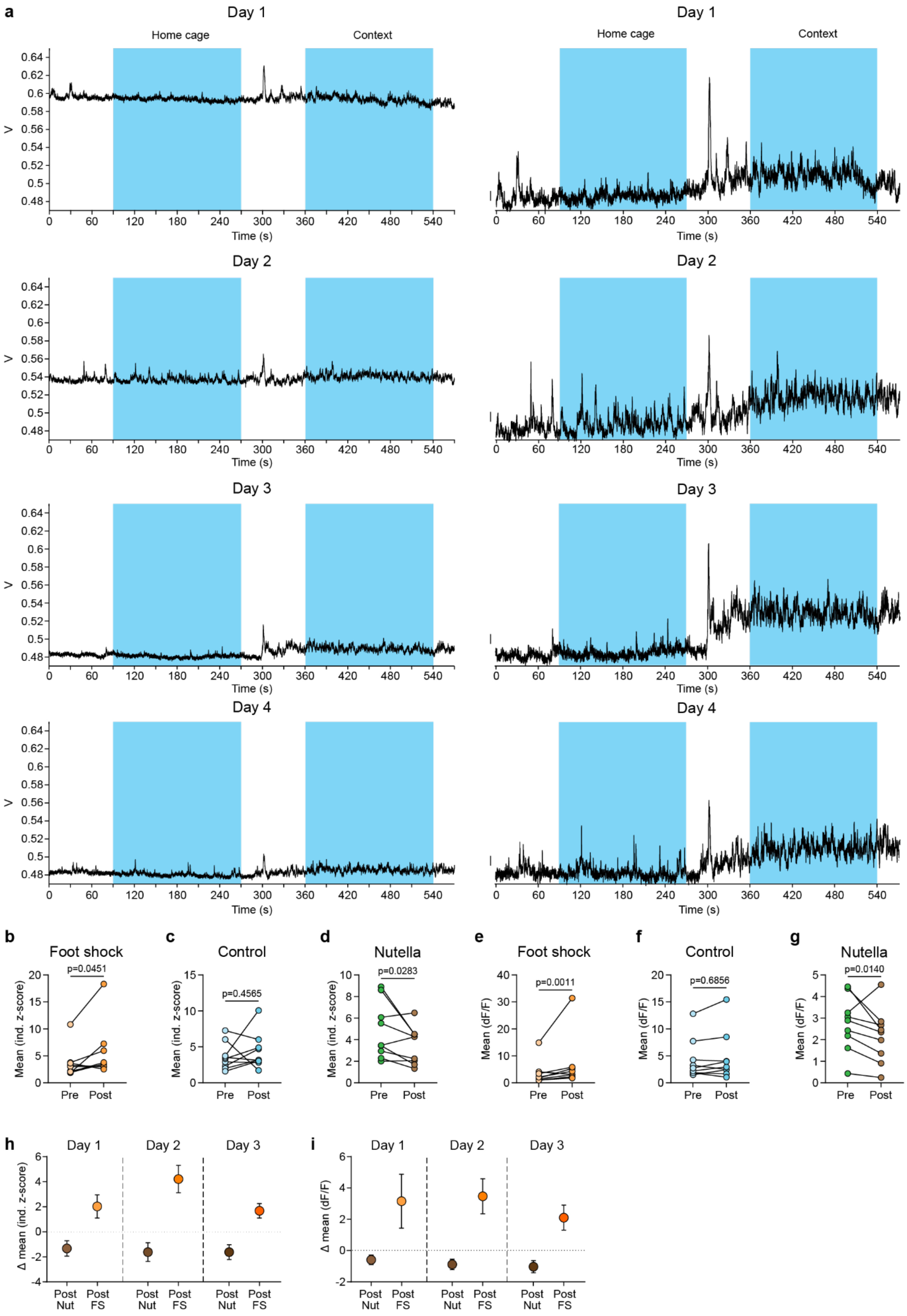
a
Day 1
Home cage
Context
V
Time (s)
Day 2
Day 3
Day 4
b
Foot shock
p=0.0451
Mean (ind. z-score)
Pre
Post
c
Control
p=0.4565
d
Nutella
p=0.0283
e
Foot shock
p=0.0011
Mean (dF/F)
f
Control
p=0.6856
g
Nutella
p=0.0140
h
Day 1
Day 2
Day 3
Δ mean (ind. z-score)
Post Nut
Post FS
i
Δ mean (dF/F)

Supplementary Fig. 10. Photometry recordings on consecutive days.

a, Illustrative fiber photometry traces of repeated context exposures on consecutive days. Raw traces on the left column, bleaching and motion corrected z-score calculated counterparts are on the right. Highlighted segments are used for further analysis. **b**-**d**, Results of photometry data analyses using z-score calculated independently from other experiments (ind. z-score) before and after foot shock (**b**, p=0.0451, n=9 mice, t=2.372, ratio paired two-tailed t-test), control exposure (**c**, p=0.4565, n=9 mice, t=0.7824, ratio paired two-tailed t-test) and Nutella (**d**, p=0.0283, n=9 mice, t=2.672, ratio paired two-tailed t-test). **e**-**g**, Results of photometry data analyses using dF/F calculations before and after foot shock (**e**, p=0.0011, n=9 mice, t=4.990, ratio paired two-tailed t-test), control exposure (**f**, p=0.6856, n=9 mice, t=0.4199, ratio paired two-tailed t-test) and Nutella (**g**, p=0.0140, n=9 mice, t=3.131, ratio paired two-tailed t-test). **h**, Relative changes of population activity recorded using single fiber photometry during all re-exposures to the hazelnut context (Nut, $Post_{Day\ 1}$ - $Post_{Day\ 3}$: P=0.6717, F(2,16)=0.4081, mixed-effects model) and foot shock context (FS, $Post_{Day\ 1}$ - $Post_{Day\ 3}$: P=0.2875, F(2,26)=1.308, mixed-effects model) using z-score calculated independently from other experiments (ind. z-score). Comparison of the valence effects (Nut vs FS: P=0.0002, F(1,16)=22.11, mixed-effects 2-way ANOVA). **i**, Relative changes of population activity recorded using single fiber photometry during all re-exposures to the hazelnut context (Nut, $Post_{Day\ 1}$ - $Post_{Day\ 3}$: P=0.0611, F(2,16)=3.347, mixed-effects model) and foot shock context (FS, $Post_{Day\ 1}$ - $Post_{Day\ 3}$: P=0.9868, F(2,26)=0.0133, mixed-effects model) using dF/F calculations. Comparison of the valence effects (Nut vs FS: P=0.0074, F(1,16)=9.402, mixed-effects 2-way ANOVA). Scale bars, **a**, 2 z-score.

Supplementary Tables

Figure panel	Groups	dimension	P	n	t / F	test
Fig 1e	HC: 0.05 ± 0.1<br>NC: 3.16 ± 0.51	z-score	0.0001	10 mice	t=6.34	paired two-tailed t-test
Fig 1g	HC: 0.41 ± 0.02<br>NC: 0.92 ± 0.03	idF/F	1.057×10^{-17}	96 cells, 4 mice	t=10.56	paired two-tailed t-test
Fig 2b	Pre: 2.96 ± 0.34<br>Post: 4.69 ± 0.67	z-score	0.0079	9 mice	t=3.514	paired two-tailed t-test
Fig 2d	Pre: 0.69 ± 0.02<br>Post: 1.01 ± 0.03	idF/F	6.634×10^{-16}	115 cells, 5 mice	t=9.409	paired two-tailed t-test
Fig 2f	Pre: 3.32 ± 0.54<br>Post: 3.23 ± 0.43	z-score	0.7772	9 mice	t=0.2927	paired two-tailed t-test
Fig 2h	Pre: 0.92 ± 0.03<br>Post: 0.93 ± 0.03	idF/F	0.576	96 cells, 4 mice	t=0.5612	paired two-tailed t-test
Fig 2k Weak	Pre: 0.44 ± 0.02<br>Post: 0.96 ± 0.07	idF/F	2.952×10^{-10}	37 cells	t=8.602	paired two-tailed t-test
Fig 2k Strong	Pre: 1.06 ± 0.03<br>Post: 1.05 ± 0.07	idF/F	0.9748	17 cells	t=0.032	paired two-tailed t-test
Fig 2k Post	Weak: 0.96 ± 0.07<br>Strong: 1.05 ± 0.07	idF/F	0.4115	37 cells<br>17 cells	t=0.8279	two-tailed t-test
Fig 2n Weak	Pre: 0.44 ± 0.04<br>Post: 0.56 ± 0.07	idF/F	0.0253	15 cells	t=2.504	paired two-tailed t-test
Fig 2n Strong	Pre: 1.27 ± 0.03<br>Post: 1.12 ± 0.04	idF/F	0.0019	28 cells	t=3.45	paired two-tailed t-test
Fig 2n Post	Weak: 0.56 ± 0.07<br>Strong: 1.12 ± 0.04	idF/F	2.670×10^{-05}	15 cells<br>28 cells	t=7.617	two-tailed t-test
Fig 2o	Weak: 1.54 ± 0.21<br>Strong: 0.71 ± 0.12	idF/F	0.0156	37 cells<br>17 cells	t=2.501	two-tailed t-test
Fig 4b	$Post_{Day\ 1}$: 69.1 ± 8.6<br>$Post_{Day\ 2}$: 20.8 ± 4.9<br>$Post_{Day\ 3}$: 8.7 ± 3.6	s	5.638×10^{-10}	15 mice	F(2,28)=50.09	repeated measures 1-way ANOVA

Fig 4c	$Post_{Day\ 1}$: 151.8 ± 8.1	ng/ml	0.3655	8 mice	F(2,14)=1.082	repeated measures 1-way ANOVA
	$Post_{Day\ 2}$: 171.5 ± 10.3					
	$Post_{Day\ 3}$: 147.6 ± 15.7					
Fig 4e	$Post_{Day\ 1}$: 4.88 ± 0.50	z-score	0.2722	13 mice	F(2,26)=1.369	mixed effects model
	$Post_{Day\ 2}$: 5.61 ± 0.56			15 mice		
	$Post_{Day\ 3}$: 4.94 ± 0.42			16 mice		
Fig 4f	$Post_{Day\ 1}$: 1.01 ± 0.03	idF/F	0.1003	115 cells, 5 mice	F(2,228)=2.323	repeated measures 1-way ANOVA
	$Post_{Day\ 2}$: 0.96 ± 0.03					
	$Post_{Day\ 3}$: 0.99 ± 0.03					
Fig 5b	Pre: 3.77 ± 0.52	z-score	0.0116	9 mice	t=3.257	paired two-tailed t-test
	Post: 2.65 ± 0.46					
Fig 5d	Pre: 4.64 ± 0.42	idF/F	0.0011	90 cells, 5 mice	t=3.362	paired two-tailed t-test
	Post: 3.58 ± 0.34					
Fig 5g Weak	Pre: 2.14 ± 0.13	idF/F	0.6368	51 cells	t=0.4751	paired two-tailed t-test
	Post: 2.27 ± 0.26					
Fig 5g Strong	Pre: 11.09 ± 1.07	idF/F	0.0063	17 cells	t=3.144	paired two-tailed t-test
	Post: 7.49 ± 1.18					
Fig 5h Nut	$Post_{Day\ 1}$: -1.13 ± 0.35	z-score	0.3302	9 mice	F(2,16)=0.1188	repeated measures 1-way ANOVA
	$Post_{Day\ 2}$: -1.39 ± 0.35					
	$Post_{Day\ 3}$: -1.48 ± 0.36					
Fig 5h FS	$Post_{Day\ 1}$: 1.74 ± 0.49	z-score	2.063×10^{-06}	9 mice	F(1,16)=52.05	2-way ANOVA
	$Post_{Day\ 2}$: 2.38 ± 0.52					
	$Post_{Day\ 3}$: 1.49 ± 0.44					
Fig 5i	$Post_{Day\ 1}$: 3.58 ± 0.35	idF/F	0.055	90 cells, 5 mice	F(2,178)=5.368	repeated measures 1-way ANOVA
	$Post_{Day\ 2}$: 4.00 ± 0.37					
	$Post_{Day\ 3}$: 3.25 ± 0.30					
			$Post_{Day\ 1}$ vs $Post_{Day\ 3}$: 0.4470			Bonferroni’s multiple comparisons
Supp. Fig 2b	HC#1: 6.66 ± 0.25	idF/F	0.1682	116 cells, 4 mice	t=0.1387	paired two-tailed t-test
	HC#2: 6.45 ± 0.22					
Supp. Fig 3a	Pre: 0.70 ± 0.03	z-score	0.0246	5 mice	t=3.515	paired two-tailed t-test
	Post: 1.03 ± 0.07					
Supp. Fig 3c			0.6505	9 mice	F(9,72)=0.7630	repeated measures 1-way ANOVA
Supp. Fig 3d	NC Post: 114.0 ± 19.5	ng/ml	0.0002	6 mice	t=5.276	two-tailed t-test
	FS Post: 224.7 ± 11.3			9 mice		

Supp. Fig 4b Weak	Pre: 0.33 ± 0.03<br>Post: 0.78 ± 0.10	idF/F	1.076x10^{-4}	16 cells	t=5.201	paired two-tailed t-test
Supp. Fig 4b Strong	Pre: 1.06 ± 0.03<br>Post: 1.05 ± 0.07	idF/F	0.9748	17 cells	t=0.032	paired two-tailed t-test
Supp. Fig 4b Post	Weak Post: 0.78 ± 0.10<br>Strong Post: 1.05 ± 0.07	idF/F	0.0419	16 cells<br>17 cells	t=2.122	two-tailed t-test
Supp. Fig 6a	$\text{Post}_{\text{Day 1}}$: 1.03 ± 0.07<br>$\text{Post}_{\text{Day 2}}$: 0.98 ± 0.05<br>$\text{Post}_{\text{Day 3}}$: 1.00 ± 0.06	idF/F	0.4541	5 mice	F(2,8)=0.8726	repeated measures 1-way ANOVA
Supp. Fig 7c	Pre: 3.77 ± 0.52<br>Nut: 2.69 ± 0.45	z-score	0.0011	9 mice	t=4.952	paired two-tailed t-test
Supp. Fig 9a			0.2015	4-5 vs 5-7 mice	F(1,10)=1.870	mixed-effects 2-way ANOVA
Supp. Fig 10b	Pre: 3.61 ± 0.94<br>Post: 5.64 ± 1.67	z-score	0.0451	9 mice	t=2.372	ratio paired two-tailed t-test
Supp. Fig 10c	Pre: 3.65 ± 0.62<br>Post: 4.40 ± 0.83	z-score	0.4565	9 mice	t=0.7824	ratio paired two-tailed t-test
Supp. Fig 10d	Pre: 4.81 ± 0.87<br>Post: 3.48 ± 0.57	z-score	0.0283	9 mice	t=2.672	ratio paired two-tailed t-test
Supp. Fig 10e	Pre: 3.42 ± 1.48<br>Post: 6.57 ± 3.14	z-score	0.0011	9 mice	t=4.99	ratio paired two-tailed t-test
Supp. Fig 10f	Pre: 4.24 ± 1.25<br>Post: 4.72 ± 1.52	z-score	0.6856	9 mice	t=0.4199	ratio paired two-tailed t-test
Supp. Fig 10g	Pre: 2.74 ± 0.43<br>Post: 2.15 ± 0.42	z-score	0.014	9 mice	t=3.131	ratio paired two-tailed t-test
Supp. Fig 10h			0.0002		F(1,16)=22.11	mixed-effects 2-way ANOVA
Supp. Fig 10i Nut			0.0611		F(2,16)=3.347	mixed effects model
Supp. Fig 10i FS			0.9868		F(2,26)=0.0133	mixed effects model
Supp. Fig 10i Nut vs FS			0.0074		F(1,16)=9.402	mixed-effects 2-way ANOVA

Supplementary Table 1. Statistical details regarding each analysis.
