# Supplementary material for: Hypothalamic CRH neurons represent physiological memory of positive and negative experience
Source: Nat Commun. 2023 Dec 21;14:8522. doi: 10.1038/s41467-023-44163-5 (PMC10739955; doi:10.1038/s41467-023-44163-5)
Supplement: Supplementary file 3 — Reporting Summary [file 41467_2023_44163_MOESM3_ESM.pdf]

nature portfolio

Corresponding author(s): Jaideep S Bains

Last updated by author(s): Jan 10, 2023

Reporting Summary

Nature Portfolio wishes to improve the reproducibility of the work that we publish. This form provides structure for consistency and transparency in reporting. For further information on Nature Portfolio policies, see our Editorial Policies and the Editorial Policy Checklist.

Statistics

For all statistical analyses, confirm that the following items are present in the figure legend, table legend, main text, or Methods section.

n/a	Confirmed	
☐	☒	The exact sample size (*n*) for each experimental group/condition, given as a discrete number and unit of measurement
☐	☒	A statement on whether measurements were taken from distinct samples or whether the same sample was measured repeatedly
☐	☒	The statistical test(s) used AND whether they are one- or two-sided<br>*Only common tests should be described solely by name; describe more complex techniques in the Methods section.*
☒	☐	A description of all covariates tested
☐	☒	A description of any assumptions or corrections, such as tests of normality and adjustment for multiple comparisons
☐	☒	A full description of the statistical parameters including central tendency (e.g. means) or other basic estimates (e.g. regression coefficient) AND variation (e.g. standard deviation) or associated estimates of uncertainty (e.g. confidence intervals)
☐	☒	For null hypothesis testing, the test statistic (e.g. *F*, *t*, *r*) with confidence intervals, effect sizes, degrees of freedom and *P* value noted<br>*Give P values as exact values whenever suitable.*
☒	☐	For Bayesian analysis, information on the choice of priors and Markov chain Monte Carlo settings
☒	☐	For hierarchical and complex designs, identification of the appropriate level for tests and full reporting of outcomes
☐	☒	Estimates of effect sizes (e.g. Cohen's *d*, Pearson's *r*), indicating how they were calculated

Our web collection on statistics for biologists contains articles on many of the points above.

Software and code

Policy information about availability of computer code

Data collection	TDT Synapse (version 84-34769P, Tucker-Davis Technology) and DORIC Studio software (DORIC Lenses) software were used for the fiber photometry data collection. Inscopix IDAS was used for miniature microscopy data collection
Data analysis	Matlab 2019b was used to analyse fiber photometry data (https://github.com/leomol/FPA) and miniature microscopy data (Min1pipe, Lu et al., Cell Reports, 2018). For animal tracking: https://github.com/kpc-simone/dmbs-tracking. For behavioral annotation the same method was used as published in Fuzesi et al. Nat Comm, 2016)

For manuscripts utilizing custom algorithms or software that are central to the research but not yet described in published literature, software must be made available to editors and reviewers. We strongly encourage code deposition in a community repository (e.g. GitHub). See the Nature Portfolio guidelines for submitting code & software for further information.

Data

Policy information about availability of data

All manuscripts must include a data availability statement. This statement should provide the following information, where applicable:

- Accession codes, unique identifiers, or web links for publicly available datasets
- A description of any restrictions on data availability
- For clinical datasets or third party data, please ensure that the statement adheres to our policy

All relevant data and analysis tools are available upon reasonable request from the authors.

Human research participants

Policy information about studies involving human research participants and Sex and Gender in Research.

Reporting on sex and gender	n/a
Population characteristics	n/a
Recruitment	n/a
Ethics oversight	n/a

Note that full information on the approval of the study protocol must also be provided in the manuscript.

Field-specific reporting

Please select the one below that is the best fit for your research. If you are not sure, read the appropriate sections before making your selection.

☒ Life sciences ☐ Behavioural & social sciences ☐ Ecological, evolutionary & environmental sciences

For a reference copy of the document with all sections, see nature.com/documents/nr-reporting-summary-flat.pdf

Life sciences study design

All studies must disclose on these points even when the disclosure is negative.

Sample size	Sample sizes were based on previous experiments conducted by us and other labs using similar techniques.
Data exclusions	For fiber photometry and miniature microscopy data, mice were excluded if no calcium signal was observed in PVN CRH neurons during pick-up of the animal. We have previously shown (Daviu et al Nat Nsci, 2020) that PCN CRH neurons show a robust increase in calcium during approach and pick up.
Replication	Our observations that PVN CRH neuron activity is linked to context exposures has been reproduced in multiples cohorts of mice. The experiments have been conducted by multiple individuals in the lab.
Randomization	Mice were randomly selected. If multiple mice were used from one litter the mice were spread across experimental groups to avoid a litter effect.
Blinding	Due to the nature of the experiments, one experimenter overseeing the experiment could not be blind to the experimental groups. Experimenter interference was minimalized during behavior recordings.

Reporting for specific materials, systems and methods

We require information from authors about some types of materials, experimental systems and methods used in many studies. Here, indicate whether each material, system or method listed is relevant to your study. If you are not sure if a list item applies to your research, read the appropriate section before selecting a response.

Materials & experimental systems

n/a	Involved in the study	
☒	☐	Antibodies
☒	☐	Eukaryotic cell lines
☒	☐	Palaeontology and archaeology
☐	☒	Animals and other organisms
☒	☐	Clinical data
☒	☐	Dual use research of concern

Methods

n/a	Involved in the study	
☒	☐	ChIP-seq
☒	☐	Flow cytometry
☒	☐	MRI-based neuroimaging

Animals and other research organisms

Policy information about studies involving animals; ARRIVE guidelines recommended for reporting animal research, and Sex and Gender in Research

Laboratory animals	For fiber photometry and behavioral tracking experiments Crh-IRES-Cre (B6(Cg)-Crhtm1(cre)Zjh/J; stock number 012704) and Ai14 (B6.Cg-Gt(ROSA)26Sortm14(CAG-TdTomato)Hze/J; stock number 007914) mice in which CRH neurons express tdTomato fluorophore were used. For some fiber photometry and all of the miniature microscopy experiments the offsprings of Crh-IRES-Cre mice were crossed with Ai148 (Ai148(TIT2L-GC6f-ICL-tTA2)-D; stock number 030328) animals were utilized.
Wild animals	n/a
Reporting on sex	Male and female mice were used in this study. Initial analyses showed no difference between sexes and the low number of animals used in miniature microscopy recordings make sex based separation statistically underpowered.
Field-collected samples	n/a
Ethics oversight	All animal protocols were approved by the University of Calgary Animal Care and Use Committee (AC21-0097).

Note that full information on the approval of the study protocol must also be provided in the manuscript.
